# Learning from complex elderly care: a qualitative study on motivating residents in family medicine

**DOI:** 10.1186/s12875-022-01908-3

**Published:** 2022-12-01

**Authors:** K. W.J. Koetsenruijter, W. Veldhuijzen, J. De Lepeleire, Y van Leeuwen, J. W.M. Muris, P. W. Teunissen

**Affiliations:** 1 Department of Family Medicine, Maastricht University, Maastricht, the Netherlands; 2 School of Health Professions Education (SHE), Maastricht University, Maastricht, the Netherlands; 3 Department of Public Health and Primary Care, KU Leuven, Leuven, Belgium; 4 Department of Family Medicine and Care and Public Health Research Institute (CAPHRI), Maastricht University, Maastricht, the Netherlands; 5 School of Health Professions Education (SHE), Department of Obstetrics and Gynecology, Maastricht University Medical Center +, Maastricht, the Netherlands

**Keywords:** Motivation, Elderly care, Medical residency, Self-determination theory, Complex care

## Abstract

**Background:**

More and more patients need complex care, especially the elderly. For various reasons, this is becoming increasingly difficult. The onus is essentially on family physicians to provide this care and family medicine residency programs should therefore prepare their residents for this task. We know from self-determination theory (SDT) that motivation plays a key role in learning and that in order to boost motivation, fulfillment of 3 basic psychological needs - for autonomy, competence, and relatedness – is crucial. As residents often lack motivation, residency programs face the important challenge to motivate them to learn about and engage in complex elderly care. How to do so, however, is not yet sufficiently understood.

**Methods:**

We conducted a qualitative multi-institutional case study across four universities in Belgium and the Netherlands. In the period between June, 2015, and May, 2019, we triangulated information from semi-structured interviews, document analysis, and observations of educational moments. Guided by SDT concepts, the analysis was performed iteratively by a multidisciplinary team, using ATLAS.ti, version 8. In this process, we gained more insights into residents’ motivation to learn complex elderly care.

**Results:**

We scrutinized 1,369 document pages and 4 films, observed 34 educational moments, and held 41 semi-structured interviews. Although we found all the 3 basic psychological needs postulated by SDT, each seemed to have its own challenges. First, a tension between the need to guide residents and to encourage their independent learning complicated fulfillment of the need for autonomy. Second, the unpredictability of complex care led to reduced feelings of competence. Yet, guidelines and models could help residents to capture and apprehend its complexity. And third, family medicine practice, patients, and educational practice, by either satisfying or thwarting the need for relatedness, were identified as key mediators of motivation. By setting the right example and encouraging residents to discuss authentic dilemmas and switch their health care approach from cure to care, educators can boost their motivation.

**Conclusion:**

Our study has demonstrated that the degree of perceived autonomy, guidance by the education program, use of authentic dilemmas, as well as involvement of group facilitators can aid the process of motivation.

**Trial registration:**

NVMO, ERB number 482.

**Supplementary Information:**

The online version contains supplementary material available at 10.1186/s12875-022-01908-3.

## Introduction

Aging of the population means that more care will be needed in the near future. Not only will the number of consultations rise, but health professionals will also have to deal with health problems that are becoming increasingly complex [1]. Considered a key driver of the demand for health services [2], complex elderly care typically involves one or more of the following components: “chronic conditions and multiple morbidities, cognitive and/or mental problems, nursing and/or medical treatment, coordination of formal health care services, collaborative interdisciplinary approaches and home care supports” [3]. Factors contributing to the complexity of the problems in elderly care include the closure of homecare facilities, less qualified staff, enhanced therapeutic options, and multi-morbidity. Family physicians must deal with such problems on a daily basis. A study of primary care physicians by Adams et al. has shown that providing complex elderly care is difficult indeed [4]. What also complicates the provision of elderly care is the fact that family physicians must take a different approach to health care. Whereas primary health care often takes a reactive approach to the treatment of health problems, the elderly population requires a proactive approach. Consequently, family physicians must adopt a different way of thinking and approaching health issues [5, 6]. Together, the above factors make complex elderly care more difficult to deliver than the standard care that family physicians are used to. It is therefore imperative that residents in family medicine learn how to provide this complex care.

Perhaps the greatest challenge of training future physicians to deliver complex elderly care is to motivate them for this part of their future job [7, 8]. A study by Ortolon [9] found that elderly care is less attractive to doctors than is care for younger patients. This lack of interest in elderly care can probably be explained by the following overlapping and interacting aspects: Problems are medically complex, patients with polypharmacy and multi-morbidity risk experiencing side effects when medication is added, personal and interpersonal challenges, and the burden of administration associated with elderly care [4]. Moreover, as residents often feel that elderly care patients cannot be cured, delivering such care can be less satisfying and even frustrating [8, 10]. Already in 2012, Higashi et all. were keen to point out that residents typically experience elderly care as boring and frustrating [11]. The challenge of education lies in reducing this frustration and providing residents with ways to get to grips with these complexities.

That said, a focus on residents’ motivation seems vital to meet the challenge of training residents appropriately in this care, as motivation is a prerequisite for becoming competent in this discipline. In 1938, Murray was one of the first to write about the necessity of motivation in his “need-to-achieve theory.” More recently, the importance of motivation in learning was further developed, for instance in self-determination theory (SDT) [12]. Having been cited and used frequently, this theory postulates that three basic psychological needs help optimize motivation: the need for autonomy, competence, and relatedness. Table 1 gives a more detailed description of each of these needs.

**Table 1:** Definitions of the basic psychological needs postulated by SDT [13]

Autonomy	Reflects the experience that behavior is an expression of the self and generates a complete feeling of free will, also called volition, to choose whatever a person desires or considers useful to do
Competence	To fulfill the need for competence, individuals must constantly search for and deal with challenges so as to optimize their skills and capacities
Relatedness	Reflects the desire to feel connected with others, to care and be cared for, and to have a sense of belongingness, both with significant other individuals as well as with a significant community. Rather than being a formal membership of a group or a relationship, relatedness is intended to be a psychological construct

When translating these concepts to education, tensions arise across and within the said needs. To boost residents’ motivation, educational activities ideally should not interfere too much with their sense of autonomy, competence, and relatedness. However, studies have shown that without such involvement, residents are much less exposed to elderly care than their supervisors [14, 15]. This may cause them to face less challenges and, consequently, to lose their motivation. Finding the right balance seems therefore key [11].

Little is known about how education is currently motivating residents in family medicine to learn about complex care of elderly patients. In their 2016 systematic review on using educational interventions to empower nursing home residents, Schoberer et al. concluded that motivation indeed supports empowerment. However, they did not elaborate on how motivation is created [16]. To understand how we can best prepare residents for providing such care, we need an in-depth analysis of the educational approaches currently used to motivate students to learn about complex elderly care. This gap in knowledge about how to motivate the said residents to provide complex elderly care led us to address the following research question: “How do current educational approaches affect the motivation of residents in family medicine to learn to provide complex elderly care?” We conducted a multi-institutional case study to answer this question.

## Methods

This research was a multi-institutional case study in the field of family medicine residency, with a special focus on complex elderly care. We performed the study in the period between June, 2015, and May, 2019. The family medicine residencies in both the Netherlands and Belgium have developed specific education programs in complex elderly care. We deliberately combined information from different countries and multiple universities to enrich our understanding of how residents in family medicine are motivated to learn about complex elderly care. To this end, we triangulated information from different data sources including document analysis, ethnographic observations of educational moments during the elderly care curriculum, and interviews with those involved in the educational process.

### Setting

We conducted this study in the context of family medicine residency in both the Netherlands (Universities of Nijmegen, Maastricht, Rotterdam) and Belgium (University of Leuven). We chose these universities because they all had a central position in organizing elderly care. In both countries, family doctors provide most of the complex elderly care in patients’ home setting, while often being supported by formal and informal health care providers (for more details, please see Maarse et al. [17]). All universities are responsible for organizing family medicine residencies together with their regional family medicine practices where workplace learning is situated. In the Netherlands, the family medicine residency program takes three years. In the third year of this program, residents learn how to provide complex care, especially to the elderly. Residents return to university once a week to attend a formal educational session affording them the opportunity to discuss and reflect on their experiences of elderly care. At the time this study was conducted, students in Belgium were trained to become a family physician already in their final year of medical school and they continued this training for two years thereafter. Nowadays, however, students first complete a 6-year master’s program before embarking on a 3-year post-master’s residency program in family medicine. Especially during the last year of medical school, special attention is paid to elderly care through dedicated lectures. We selected the above-listed family medicine residency programs because they had developed specific education programs on complex elderly care. More specifically, all the said universities offered additional classes on topics related to complex elderly care, gave specific assignments, or created teaching programs together with other residency programs. By combining all these different initiatives, we obtained a rich data set on different approaches to motivating residents in family medicine for elderly care.

### Data collection

#### Documents

Together with the person responsible for teaching elderly care at each respective university, KK selected all the relevant documents. To this end, she perused the electronic learning environment and selected all the documents related to the topic under scrutiny. We subsequently printed all related documents that were available to teachers, supervisors, developers, as well as residents. Either KK, WV, or YvL independently coded all documents.

#### Observations

KK and a representative from each university identified all core moments in the family medicine residency programs of the participating institutions. These moments were occasions in which family medicine residents were trained in the field of elderly care by university staff. Data were collected in the year in which elderly care was formally taught during family medicine education. After all participants had given their informed consent, we audiotaped the sessions observed. During these ethnographic observations, we made field notes while focusing on the interaction between residents and between residents and teachers. After each session, KK recorded her reflections on the respective teaching session in a research diary.

#### Interviews

We held semi-structured, in-depth interviews using the critical incident technique across five different groups of participants (see Table 2). In our case, the “critical incidents” were positive learning experiences, which means that we asked participants to elaborate on the experience itself and whatever had supported it. These could be personal learning experiences and experiences observed by faculty. When appropriate, the interviewer asked follow-up questions about how earlier experience, life experience, other educational experiences, and experiences in clinical practice had impacted on these positive learning experiences. We also collected background information on all interviewees.

**Table 2:** Explanation of the participant groups and their code names

Residents in family medicine (RFM)	Junior doctors who do residency to become a family physician
Early-career family medicine physicians (ex-RFM)	Physicians who completed their family medicine residency in 1–5 years
Group facilitators (GF)	Teachers of different backgrounds (mostly psychology or family medicine) who lead training sessions at the university
Family medicine supervisors (FMS)	Family physicians who are supervising residents during daily practice
Family medicine curriculum developers (FMCD)	Residency faculty and staff who are involved not only in teaching, but also in developing the residency program

We chose these groups because they all participated in education on complex elderly care. Early-career family physicians were included because they could reflect on what had motivated them during their residency. One interviewer (KK) conducted the interviews, which were audiotaped and subsequently transcribed verbatim. The number of interviews and variation between universities depended on whether new topics emerged during the analysis. This meant that we continued interviewing until no new themes emerged.

### Data analysis

Analysis was done in an iterative process. All members of the research team (KK, YvL, WV, JM, PT) took part in the analysis. Many different content perspectives were represented in this team, as it included a resident in family medicine (KK), family physician (JM), medical education expert, psychiatrist, and former family physician (WV), academic teacher (YvL), and an expert in workplace learning in health care (PT). Using these different perspectives increased the scope of analysis and helped to deepen our understanding of the data [18].

We used ATLAS.ti, version 8, to analyze all data. Our thematic analysis was based on both deductive and inductive coding. First, we used the three basic psychological needs postulated by SDT (see Table 1) to guide our deductive analysis. To allow for the emergence of unexpected themes, we subsequently subjected the data to inductive coding. During this phase, we sorted the codes and organized them into thematic groups. From these groups we constructed thematic frameworks that offered an overview of and visualized the aspects that play a role in motivating residents in family medicine in complex elderly care. When the frameworks became tangible, we again deductively coded the data to identify auxiliary excerpts. During team meetings, we discussed the thematic frameworks until we reached consensus (KK, WV, YvL, JM and PT).

### Ethics approval and informed consent

We obtained ethics approval for this qualitative study from the Netherlands Association for Medical Education (NVMO), ERB file number 482. All participants who took part in the educational sessions we observed and in the interviews gave their written or oral informed consent. We anonymized all data using codes.

## Results

In the next paragraphs, we will first describe the dataset and then move on to present the results that flow from our scrutiny of the data.

### Dataset

#### Documents

We included a total of 149 different documents, encompassing 1,369 pages that were drawn from different types of documents and four films (see Table 3).

**Table 3:** Characteristics of the educational resources included

Total of documents	Type of document	Number of pages
149	Text documents Articles Outcome descriptions Guidelines Presentations *TOTAL*	120 118 35 488 608 *1,369 pages*
	Films	4

#### Observations

KK observed 34 key moments in total (see Table 4). A second researcher (YvL) also attended one third of these moments. Whereas the number of participating residents mostly ranged from 10 to 18 persons, nine core moments were attended by 30–65 residents. These included national interactive educational sessions in the Netherlands, as well as lectures at the Universities of Leuven and Nijmegen. We observed significantly more core moments at the University of Nijmegen because its residency offered a four-day program rather than single sessions.

**Table 4:** Characteristics of educational core moments

	No. of observations	No. of residents per group
National education (in the Netherlands)	4	18–50
Leuven	2	30–40
Maastricht	5	10–14
Nijmegen	17	12–65
Rotterdam	6	8–18
*TOTAL*	*34*	*8–65*

#### Interviews

We held a total of 41 interviews across the five groups previously specified. Specification is found in Table 5.

**Table 5:** Interview characteristics

	Number	Age range	Male	Female
Residents in family medicine (RFM)	10	27–33		
Early-career family medicine physicians (ex-RFM)	5	32–48		
Family medicine supervisors (FMS)	9	42–61		
Group facilitators (GF)	10	41–61		
Family medicine curriculum developers (FMCD)	7	50–65		
*TOTAL*	*41*	*27–65*	*15*	*26*

### The SDT trias of motivation

Our research data revealed that specific aspects of the three basic psychological needs postulated by SDT were already present in the education programs under scrutiny. Yet, the use of SDT as a possible theoretical framework or motivational technique was not explicitly mentioned in curriculum documents on complex elderly care. In the following, we will describe the results pertinent to each respective need, starting with the need for autonomy, and then followed by competence and relatedness. To support these results, we have included representative excerpts from the data. We gave these excerpts code names that refer to the respective participant group and their sequence of participation.

### Autonomy

Education programs in family medicine use practical assignments to guide residents in learning complex elderly care. During the interviews, residents and supervisors in family medicine described these assignments as obstacles to be overcome, such as jumping through a hoop. In other words, they considered these assignments as mere checklists of what needed to be done during residency to be ticked off. As one supervisor remarked:

*I think it should be properly balanced, what I just said, I feel the program is packed with so many compulsory components… that I sometimes think, wow, . you know, in terms of assignments and. all the things they must do and whatnot. (FMS 9)*

We noticed that residents often did not get an explanation about the assignment and its value. Because of this, residents experienced less autonomy in their own learning process. Teachers who did explain how specific assignments contributed to their development created a more positive environment for the group to fulfill these assignments. This effect was especially seen when teachers linked assignments to the delivery of better care. It was less pronounced, however, when teachers linked it to certain learning goals set by the university, for example. In addition, we found that residents’ degree of autonomy was related to the nature of the assignment and the freedom of choice they experienced during these assignments. One of the curriculum developers, for instance, remarked:

*The assignment is actually a description and a recapitulation based on the learning outcomes that specify all the things involved in becoming skilled in caring for the elderly … That is, the assignment is actually nothing more than explaining to the resident, like: Well, these are, this is what you should work on, so that the assignment makes you realize that you do want to try and find it one way or another … Yes, each resident acts on it in her own way.*

Observations showed that when residents were free to carry out the assignment as they saw fit, group dynamics was characterized by more discussion among residents with more in-depth conversations.

In addition to the guidance provided by the education program, observations showed that teachers tried to stimulate the autonomy of their residents in a number of different ways. For instance, they asked residents to formulate personal learning goals and to use these goals to adapt the education program. Another way was to put together a program that matched residents’ needs/goals. Teachers also encouraged residents to discuss personal authentic dilemmas they had encountered in patient care. This technique allowed them to connect to the problems that residents were experiencing as much as possible. By making this connection, teachers helped induce discussion, resident participation, and an extensive discussion of suggestions on how to deal with complex elderly care. In the interviews, residents indicated that they felt challenged and that the suggestions they received helped them to deal with complex problems during daily practice. As one of the former residents commented:

*“So a bit of that awareness again of how and why you acted and what should or could be done differently in a next situation, so to speak..”*

### Competence

Residents felt a desire to provide good-quality care. Partly because of this, they were motivated to learn and to make an effort during education. Both interview and observation data revealed that complex elderly care could become a considerable personal challenge for residents in two different ways. First, they found it difficult to face up to the elderly’s disability and their own inability to solve this. Learning to accept this relative inability to deal with problems proactively was challenging. Consider the following quote by one of the residents:

*“Well, at least more from the perspective of a family doctor who is eager to offer remedies. And these are, from such perspective they are family doctors, they have to provide a remedy within 10 minutes, say goodbye and then on to the next. That doesn’t work with this kind of problems.”*

This sentiment was confirmed by one of the group facilitators, who remarked:

“*Making progress often means that they have a medical solution, something of an intervention and when they are unable to offer any, it pretty much means that they cannot do anything at all and this causes irritation.”* (GF 2).

According to group facilitators and curriculum developers in family medicine, residents needed to learn how to cope with these feelings. This required a change of focus, for instance from demand-oriented toward goal-oriented care for patients, or from reactive toward proactive care. To cope with feelings of powerlessness, residents also had to learn that there were no ready-made solutions to complex problems. This included accepting the fact that guidelines did not apply or even gave contradictory recommendations when several elderly care problems occurred. A second challenge for residents in family medicine was to deal with the complexity of elderly care, which could be overwhelming. In an interview, one of the residents, for instance, said:

*What I always find difficult is that you, er, that when they have a problem, it often does not occur in isolation and that they already have multiple comorbidities or that they have a whole shopping list of medication, and that is what I often found very difficult.* (RFM 8)

It followed that residents were completely absorbed by the case and had difficulty in creating an overview. Consequently, they felt a need for formal education to support their development of basic competences and knowledge, and to provide tools and tricks to create this overview. Supervisors, curriculum developers, and residents in family medicine all agreed that it was difficult to obtain such an overview of several interacting problems in complex elderly care. Group facilitators struggled with the dilemma of how to stimulate and challenge their residents, without overwhelming them with practical suggestions. The developers, however, offered two solutions. On the one hand, they used theoretical education, such as lectures and conversation skills training, to expand residents’ knowledge and skills. On the other hand, they helped residents to structure complex elderly care problems by introducing specific methods to zoom in, unravel multiple domains, and integrate the elderly’s personal goals. By structuring all problems in a broad and detailed overview, residents learned to focus on all aspects of complex elderly patients, resulting in more control and thereby boosting their motivation.

### Relatedness

We identified three different social dimensions or communities to which residents felt a sense of relatedness, specifically: family medicine practice, patients, and education. These dimensions had a clear influence on residents’ motivation to provide complex elderly care.

#### Family medicine practice

The community in which residents spend most of their residency is family medicine practice. While providing elderly care, residents work with all kinds of different specialists, doctor’s assistants, and paramedics. All these people give insight into different aspects of complex elderly care, by offering suggestions and advice on how to handle struggles and how to provide this complex care. Serving as role models, supervisors also have an important role to play in this community. Since residents identify themselves with their supervisors, the way the latter regard complex elderly care can have a crucial impact on residents’ perception of this care. In the words of one resident:

*“It also very much depends on whether your supervisor is, er, elderly-minded, to say it like that, or whether he or she anticipates it each time and acts reactively each time, so to speak”* (RFM 3).

Hence, by setting an example for residents, supervisors who were committed and enthusiastic encouraged residents to learn about complex elderly care.

#### Patients

Another factor that motivated residents was the relationships they had with their patients and their informal caregivers. These relationships motivated them to help patients and provide good care. At the same time, however, they found it difficult to identify with these patients who were older and had complex health problems. As patients were at a different stage of life and had different expectations of life, residents sometimes struggled to understand their questions and choices. Being focused on improving patients’ health problems, they were more inclined to advise about interventions, which elderly patients could experience as stressful and burdensome. This, in turn, could spark mutual incomprehension and frustration. In the interviews, participants mentioned that if residents were able to let go of their desire to improve elderly patients’ health, they would have more mental space to focus on the real needs of these patients. As one group facilitator phrased it:

*“I will have to let go of my cure and wield my care. That is a process for which we should be equipped with a lot of tools”* (GF 1).

Making this mind switch from cure to care would allow residents to set personal goals that also suit patients and, as such, can be perceived as common goals. Group facilitators and fellow residents can support this process, by focusing on patients’ needs when discussing cases.

#### Education

From the interviews and observations we gathered that, during teaching moments, residents talked about and discussed personal experiences and cases, asked critical questions, and encouraged each other to look for solutions that helped solve practical problems. One of the group facilitators summarized this process as follows:

*“ Contributing experiences… contributing a case… “What did you experience?”… and then talk about that…. putting something forward individually, letting the group think along in hopes that, as a fellow group member, you will learn something from it too… “Hey, he or she experienced this or that, which reminds me of something I experienced myself”… and that especially the one who is contributing will draw something from it in any case…”* (GF3).

Case discussions in which residents did not just talk about the case, but also ventilated their dilemmas led to a livelier discussion and increased exchange of experiences. When group facilitators asked specific questions during such discussions, we noticed that residents became more aware of their struggles, which intensified discussion and participation by several residents. Residents also seemed more motivated to engage in the discussions and learn from them when facilitators normalized their struggles and also shared their own practical experiences.

## Discussion

In this study, we triangulated information from observations, semi-structured interviews, and document analysis through the lens of SDT and its three constituent basic psychological needs to answer the research question: How do current educational approaches affect the motivation of residents in family medicine to learn to provide complex elderly care? With respect to the first need (autonomy), we found that a tension existed between the need to stimulate learning processes through practical assignments and the pursuit of self-directed learning. Residents appeared to become more motivated when case-specific learning goals were addressed, in-depth questions about possible problems were asked, and dilemmas and struggles were raised. As for the second need (competence), residents struggled to obtain a sense of competence because they were easily overwhelmed by the multitude of aspects they had to consider. In the absence of appropriate non-conflicting guidelines, they were unable to see whether the treatment they had started was according to protocol. What also detracted from their sense of competence was their inability to solve problems. Group facilitators and curriculum developers, in their turn, sought to remediate this lack of competence by providing residents with tools to unravel complex cases. Finally, with regard to the third need (relatedness), we found that family medicine practice, patients, informal caregivers, other health care providers, and faculty and peers were important mediators. Supervisors and group facilitators who were enthusiastic, asked residents critical questions, involved them in case-based discussions, and helped unravel their dilemmas positively influenced residents’ motivation.

Considering the foregoing, we can confirm that the basic psychological needs postulated by SDT – autonomy, competence, and relatedness – are, indeed, needed for motivation [13]. Our application of these needs to the teaching of complex elderly care has offered new insights into how such education can be optimized to maximize residents’ motivation to learn this specific discipline. Our study revealed that all the said needs were already implicitly embedded in the curriculum. More specifically, supervisors and group facilitators tacitly applied them in their teaching. In 2002, Reeve [19] was already keen to underscore the importance of teacher alignment on autonomy in optimizing student motivation. In his investigation, the autonomy-supportive teaching style was flagged as the preferred approach, which included listening to and creating space for students. To these components, our study adds enthusiasm and involvement by group facilitators and supervisors in family medicine. As these actors play an important role in motivating residents to learn to provide complex elderly care, they deserve special attention as additional mediators of motivation.

Another possible way to motivate family medicine residents is to help them to manage complex care. By making complex problems manageable, residents will obtain an overview and feel less overwhelmed. Frequently used in other work fields, complexity theory has taught us that problems, when too complex, can be broken down into manageable parts [20]. Similarly, in order to experience some control, residents must zoom in on only a few aspects at a time. In our study, group facilitators and curriculum developers offered them the appropriate tools to help them do so. In this way, residents were able to keep an overview of problems and stay motivated to learn and provide complex care. Sometimes, however, this approach may still be demotivating as it may not always suffice to grasp the complexity of problems in full. To boost residents’ motivation, it is essential that they understand the full measure of complexity in elderly care. Complexity theory posits that zooming out helps to put all aspects of the context and their interdependence into perspective, to see it as a whole [20, 21]. With this knowledge in mind, we can conclude that current methods do teach residents to zoom in, but not to zoom out. We advocate for including both approaches in education, as it helps to boost residents’ motivation to deliver complex elderly care. After all, residents take more pleasure in providing complex elderly care if they feel competent to deal with complexity.

Research on elderly care has pointed out that collaboration with informal caregivers and with other health care providers is crucial for the delivery of complex elderly care [22, 23]. Our findings suggest that these care partners are also instrumental in enhancing a sense of relatedness in residents. That is to say, not only relationships with patients, but also relationships with informal caregivers can be important sources of motivation for residents [24]. These relationships could be important to learn from in context of roles of GP’s in multidisciplinary teams. Grol et al. stated in 2018 that GP’s have an indispensable role in multidisciplinary teams [25]. So, learning with and from formal and informal caregivers can be important in motivating GP residents.

### Implications for practice

Our study has demonstrated that current education programs do motivate family medicine residents to learn how to provide high-quality complex elderly care. To strengthen their motivation, however, we recommend that the elements described in the Results section be integrated more into current curricula. Discussions, taking dilemmas as a starting point, involving peers, and sharing cases can help to guide their learning process and to motivate them to undertake this learning trajectory. We identified the use of dilemmas as a “best practice,” because dilemmas can serve as a starting point for teaching all three basic psychological needs. By defining and exploring cases that family medicine residents are struggling with and taking their lived experience as a starting point, residents’ autonomy can be supported. This approach also helps to promote a sense of competence in residents and to satisfy the relational need to provide good care and to share the burden of deciding on what constitutes good care. Faculty play an important role in this process, for they can support and train residents during educational sessions in actively applying these strategies. We therefore recommend, for instance, that faculty development programs shift their focus to guiding and educating supervisors in how to deal with complexity and how to tolerate the uncertainties inherent in the provision of complex elderly care. Furthermore, to motivate residents and to keep them motivated, sometimes it seems necessary to limit their autonomy. Yet, the extent to which this is done calls for a delicate balance between setting boundaries and offering guidance on the one hand, and creating opportunities for self-development, ambitions, and choices on the other. The key to resolving this conundrum may be to let group facilitators, supervisors, and residents in family medicine share their goals at the beginning of the education program.

### Implications for research

This study has shown the ways in which family medicine residents are currently motivated to learn complex elderly care. However, we did not study the effect and role of education during working hours in family medicine practice. To enhance our understanding of the factors that influence motivation, additional observations during practice of complex elderly care could be meaningful, especially when these do not only focus on supervisors, but also on informal caretakers, patients, and other health care providers. Questions that would be of interest to explore are: What kind of techniques do supervisors and other stakeholders in family medicine use to motivate residents to provide complex elderly care? What role do they play in it? These questions are relevant because residents spend most of their residency in family medicine practice.

### Strength and limitations

One of the strengths of this study is the richness of the data. Not only did we conduct this study in two different countries and across four different universities, but we also included many different stakeholders from the family medicine residency as study participants. The study also had two limitations. The first concerns the transferability of our results. All participating universities had a special interest in complex elderly care and in motivating future family physicians to learn and provide it. This means that the curricula of the residency programs to which we had access paid specific attention to this subject. Universities that do not have a special interest in complex elderly care are likely to have different results. Another limitation is the fact that is it hard to capture the different elements that motivate residents to learn complex elderly care. Although we identified and described specific relationships, this does not necessarily imply that, once implemented, either in whole or in part, these will enlarge motivation. The complexity of the process of motivation is such that it is impossible to guarantee that the educational elements identified in this study will motivate all family medicine residents.

## Conclusion

Motivating family medicine residents to become proficient in providing complex elderly care is challenging. Our research based on triangulation of observations, interviews, and documents shows how current educational approaches in two Western European countries affect residents’ motivation. Residents appear to be overwhelmed by the complexity of problems in elderly care. Motivating them to deal with this complex care helps to learn how to provide it. Our study shows that the degree of perceived autonomy, guidance by the education program, use of authentic dilemmas, as well as involvement of group facilitators can aid the process of motivation.

## Supplementary Information

**Additional file 1:** **Appendix 1. **Document characteristics.

## Data Availability

If necessary, the datasets used and/or analyzed during the present study can be requested from the corresponding author. We chose this option because we are currently still using the same dataset to write another article. As soon as all articles that we based on this database have been published, it will be possible to make the database accessible. Note: the database consists of interviews, observations, and related notes, all in Dutch.

## Abbreviations

SDT: Self-determination theory
RFM: Residents in family medicine
ex-RFM: Early-career family medicine physicians
GF: Group facilitators
FMS: Family medicine supervisors
FMCD: Family medicine curriculum developers
