# Supplementary material for: Learning from complex elderly care: a qualitative study on motivating residents in family medicine
Source: BMC Prim Care. 2022 Dec 1;23:307. doi: 10.1186/s12875-022-01908-3 (PMC9714098; doi:10.1186/s12875-022-01908-3)
Supplement: Supplementary file 1 — Additional file 1: Appendix 1. Document characteristics. [file 12875_2022_1908_MOESM1_ESM.docx]

**Appendix 1:** Document characteristics

| University | Type of document | Number of pages |
| --- | --- | --- |
| Leuven | Text documents  Articles  Goals  Guidelines  Presentations  Films | 6  71  21  274  8  0 |
| Maastricht | Text documents  Articles  Goals  Guidelines  Presentations  Films | 2  6  0.5  70  33  4 |
| Nijmegen | Text documents  Articles  Goals  Guidelines  Presentations  Films | 94.5  17  7  8  321  0 |
| Rotterdam | Text documents  Articles  Goals  Guidelines  Presentations  Films | 17.5  24  6.5  136  246  0 |
|  | Total | 1,369 |
